# Draft Genome Sequences of Five *Proteobacteria* Isolated from Lechuguilla Cave, New Mexico, USA, and Insights into Taxonomy and Quorum Sensing

**DOI:** 10.1128/MRA.00913-19

**Published:** 2019-10-03

**Authors:** Han Ming Gan, Peter C. Wengert, Hazel A. Barton, André O. Hudson, Michael A. Savka

**Affiliations:** a Centre for Integrative Ecology, School of Life and Environmental Sciences, Deakin University, Geelong, Victoria, Australia; b Deakin Genomics Centre, Deakin University, Geelong, Victoria, Australia; c Department of Biology, University of Akron, Akron, Ohio, USA; d The Thomas H. Gosnell School of Life Sciences, Rochester Institute of Technology, Rochester, New York, USA; Indiana University, Bloomington

## Abstract

Genomic resources remain scarce for bacteria isolated from oligotrophic caves. We sequenced the genomes of five *Proteobacteria* isolated from Lechuguilla Cave in New Mexico. Genome-based phylogeny indicates that each strain belongs to a distinct genus. Two *Rhizobiaceae* isolates possess genomic potential for the biosynthesis of acyl-homoserine lactone.

## ANNOUNCEMENT

Adequate genomic resources are crucial for the understanding of adaptation for culturable microbes in nutrient-limited cave environments (1). To date, bacterial genomes isolated from caves have been poorly represented in the public databases (2–5). Here, we report the genomes of five isolates from Lechuguilla Cave in New Mexico and use genome-based phylogeny to refine their taxonomic assignment. We identified genomic potential for the biosynthesis of cell-cell communication signal in two *Rhizobiaceae* isolates and confirmed the predicted phenotype using an Agrobacterium tumefaciens reporter assay (6).

Initial isolation of the bacterial strains from remote sample sites in Lechuguilla Cave was previously described by Bhullar et al. (7). Strains were grown in half-strength tryptic soy broth with shaking at 30°C for 2 days. DNA extraction used the GenElute bacterial genomic DNA kit (MilliporeSigma, St. Louis, MO). The sequencing library was generated using the tagmentation-based Nextera XT DNA sample prep kit (Illumina, San Diego, CA) and sequenced on an Illumina MiSeq instrument (run configuration of 2 × 150 bp). The paired-end reads were adapter trimmed and assembled with Trimmomatic v0.36 (8) and Unicycler v0.4.7 (9), respectively, using the default settings.

**FIG 1:**
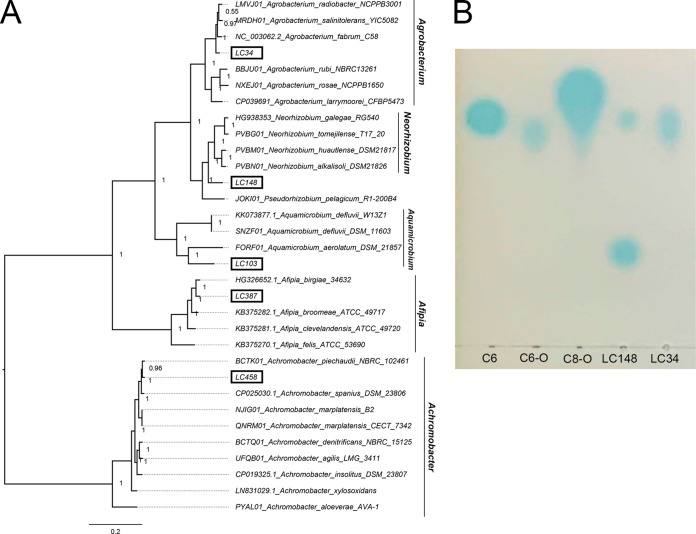
(A) Maximum likelihood tree based on the concatenated alignment of 119 conserved *Proteobacteria* single-copy gene sets generated using the default setting of GToTree v1.2.1. Branch lengths indicate the number of substitutions per site, while node labels are Shimodaira-Hasegawa (SH) local support values computed in FastTree2 (20). (B) Separation of AHL molecules from the LC34 and LC148 extracts by C_18_ reversed-phase thin-layer plate developed with methanol/water (60:40, vol/vol). Standards include the following: C6, *N*-hexanoyl-l-homoserine lactone; C6-O, *N*-(3-oxohexanoyl)-l-homoserine lactone; C8-O, *N*-(3-oxo-octanoyl)-l-homoserine lactone.

The genome of LC387 was assembled into a single contig (Table 1). Based on BLASTN alignment (10), its full-length 16S rRNA sequence is 100% identical to that of Afipia massiliensis CCUG 45153^T^ (NCBI RefSeq accession number NR_025646). For the generation of genome-based phylogeny using GToTree v1.2.1 (11), genome assemblies of bacterial type strains exhibiting high 16S rRNA gene sequence similarity to the cave isolates were downloaded and included in the pipeline. Based on phylogenomic clustering, confident taxonomic assignment to the genus level was obtained for LC34, LC103, and LC458 (Fig. 1A). The basal placement of LC148 in the Neorhizobium clade suggests that it is a divergent member within the genus or a member of an undescribed genus. Therefore, strain LC148 was classified as a Rhizobiaceae sp., pending future taxonomic investigation.

**TABLE 1:** Genome assembly metrics and data availability

BioProject accession no.	Assembly accession no.	Organism	Strain	No. of reads	No. of bases (Mb)	Estimated genome coverage (×)	Genome size (bp)	GC content (%)	No. of contigs (>500 bp)	*N*_50_ length (bp)
PRJNA281560	LBCQ00000000	Aquamicrobium sp.	LC103	1,246,649	417	69.5	6,044,022	64.06	34	330,476
PRJNA532460	SZVV00000000	*Rhizobiaceae* sp.	LC148	399,832	110	18.8	5,822,494	61.45	86	124,816
PRJNA281572	LBHX00000000	*Agrobacterium* sp.	LC34	843,163	247	46.6	5,303,297	59.35	36	377,718
PRJNA281679	LBIA00000000	Afipia massiliensis	LC387	777,637	216	48.0	4,495,198	61.35	1	4,495,198
PRJNA281681	LBHW00000000	Achromobacter sp.	LC458	1,286,382	448	68.4	6,547,009	64.55	102	143,492

We used a previously described hidden Markov model (HMM) approach (12) to identify the genomic potential for biosynthesis of acyl-homoserine lactone (AHL) molecules involved in the regulation of gene expression in response to cell density (13). AHL synthase homologs (LuxI) were identified in strains LC34 (GenBank Protein accession number TKT57503) and LC148 (GenBank Protein accession numbers TKT46115 and TKT66962). The genes coding for these proteins were similarly classified as *luxI* homologs by antiSMASH 4 and the NCBI Prokaryotic Genome Annotation Pipeline (14, 15). To confirm the predicted phenotype, LC34 and LC148 were grown in yeast mannitol (YM) medium, and ethyl acetate extracts from the cultures were tested for the presence of AHL signals using the AHL-dependent biosensor Agrobacterium tumefaciens NTL4(pZLR4) (16). LC34 produced one type of AHL signal with a retardation factor (*R_f_*) that is similar to that of 3-oxo-C8. LC148 produced two distinct AHLs, one with an *R_f_* value similar to that of C6 and the other with an *R_f_* value smaller than the included AHL standards (Fig. 1B). Future work investigating the role of quorum sensing in these cave isolates through transposon mutagenesis (17), targeted gene deletion (18), or transcriptome sequencing (19) will be informative in understanding bacteria from cave environments.

### Data availability.

The raw Illumina paired-end reads and genome assemblies have been deposited in GenBank under the BioProject numbers listed in Table 1. Bacterial strains can be requested from Michael A. Savka (Rochester Institute of Technology [RIT], NY, USA).
